# Major Histocompatibility Complex Genes Map to Two Chromosomes in an Evolutionarily Ancient Reptile, the Tuatara *Sphenodon punctatus*

**DOI:** 10.1534/g3.115.017467

**Published:** 2015-05-07

**Authors:** Hilary C. Miller, Denis O’Meally, Tariq Ezaz, Chris Amemiya, Jennifer A. Marshall-Graves, Scott Edwards

**Affiliations:** * Allan Wilson Centre for Molecular Ecology and Evolution, School of Biological Sciences, Victoria University of Wellington, Wellington 6140, New Zealand; † Comparative Genomics Group, Research School of Biological Sciences, Australian National University, Canberra ACT 0200, Australia; ‡ Molecular Genetics Program, Benaroya Research Institute at Virginia Mason, Seattle, Washington 98101; § Department of Organismic and Evolutionary Biology and Museum of Comparative Zoology, Harvard University, Cambridge, Massachusetts 02138

**Keywords:** reptilia, MHC class I, MHC class II, comparative genomics

## Abstract

Major histocompatibility complex (MHC) genes are a central component of the vertebrate immune system and usually exist in a single genomic region. However, considerable differences in MHC organization and size exist between different vertebrate lineages. Reptiles occupy a key evolutionary position for understanding how variation in MHC structure evolved in vertebrates, but information on the structure of the MHC region in reptiles is limited. In this study, we investigate the organization and cytogenetic location of MHC genes in the tuatara (*Sphenodon punctatus*), the sole extant representative of the early-diverging reptilian order Rhynchocephalia. Sequencing and mapping of 12 clones containing class I and II MHC genes from a bacterial artificial chromosome library indicated that the core MHC region is located on chromosome 13q. However, duplication and translocation of MHC genes outside of the core region was evident, because additional class I MHC genes were located on chromosome 4p. We found a total of seven class I sequences and 11 class II β sequences, with evidence for duplication and pseudogenization of genes within the tuatara lineage. The tuatara MHC is characterized by high repeat content and low gene density compared with other species and we found no antigen processing or MHC framework genes on the MHC gene-containing clones. Our findings indicate substantial differences in MHC organization in tuatara compared with mammalian and avian MHCs and highlight the dynamic nature of the MHC. Further sequencing and annotation of tuatara and other reptile MHCs will determine if the tuatara MHC is representative of nonavian reptiles in general.

Major histocompatibility complex (MHC) genes are central to the vertebrate immune response. Classical class I and II MHC genes encode cell-surface proteins that present antigens to T cells, thus facilitating self/nonself recognition. Class I molecules comprise a single polypeptide chain, encoded by a single gene, and an associated β2-microgobulin molecule, both of which are expressed on all nucleated cells and mainly present endogenous peptides to CD8+ T cells (Bjorkman and Parham 1990). By contrast, class II molecules comprise two polypeptide chains, α and β, each encoded by a separate gene, expressed only on specialized antigen-presenting cells such as B cells or macrophages, and present peptides derived from extracellular pathogens to CD4+ T helper cells (Kappes and Strominger 1988). High levels of diversity at classical class I and class II MHC genes enable organisms to counter a wide variety of pathogens (Doherty and Zinkernagel 1975). Hence, these genes are among the most polymorphic known in vertebrates and many studies have demonstrated a link between MHC variation and disease resistance or susceptibility (*e.g.*, Penn *et al.* 2002, Siddle *et al.* 2007; Alcaide *et al.* 2010).

In most vertebrates, MHC genes are located in one contiguous region of the genome. In addition to class I and class II MHC genes, this region usually also contains genes for antigen processing (*e.g.*, *TAP1*, *TAP2*, *TAPBP*), complement factors and cytokines (*e.g.*, *C2*, *C4*, and tumor necrosis factor-α), and other “framework” genes that are syntenic with the MHC in most vertebrates (Kelley *et al.* 2005). However, there are considerable differences in the arrangement of these genes and the overall size of the MHC region across different vertebrate groups.

In eutherian mammals the MHC region is large (∼4 Mb in humans) and gene dense, with a well-conserved gene order (Kelley *et al.* 2005). The class I and II regions are separated by a class III region containing cytokine and complement factor genes. The antigen processing genes *TAP1*, *TAP2*, *PSMB8*, and *PSMB9*, which process peptides for loading onto class I genes, are located in the class II region. By contrast, marsupials, monotremes, and nonmammalian vertebrates display a greater diversity in MHC organization, and the class I and II regions are rearranged compared with eutherian mammals (Kelley *et al.* 2005). In most nonmammalian vertebrates studied thus far, class I and II genes are located adjacent to one another with no intervening class III region, and the class I antigen processing genes are located within the class I region (Kaufman *et al.* 1999; Ohta *et al.* 2006). This arrangement also has been found in marsupials, suggesting that it may represent an ancestral MHC organization (Belov *et al.* 2006).

The chicken MHC (B-complex), the first bird MHC to be sequenced, revealed some striking differences in MHC structure between birds and mammals (Kaufman *et al.* 1999). In addition to the rearrangement of class I and II regions, the chicken MHC is small and streamlined compared with the mammalian MHC, spanning only a few hundred kilobases. It contains fewer, smaller, and more densely packed genes than in mammals, with few repetitive elements and no pseudogenes. This “minimal essential” MHC structure for chicken (Kaufman *et al.* 1999), with its lack of redundancy and tight linkage between genes, may have important implications for the role of the MHC in disease resistance, because it results in much stronger associations between particular MHC genotypes and disease resistance or susceptibility (Kaufman 2000, 2013).

However, analyses of MHC genes in other birds show that the chicken MHC may not be typical for birds. Early genomic and transcriptomic studies of MHC genes in songbirds suggested a lower density and greater number of genes than found in chicken (Westerdahl *et al.* 1999; Gasper *et al.* 2001). The quail MHC is approximately twice the size of the chicken B-complex and the class I, class II, *NK*, *lectin*, and *B-G* genes have undergone extensive duplication (Shiina *et al.* 2004). The zebrafinch MHC occupies an even larger genomic region, being spread across at least seven bacterial artificial chromosome (BAC) clones spanning 739 kb and containing multiple class I and II genes and several pseudogenes (Balakrishnan *et al.* 2010). Thus, bird MHCs clearly show extensive lineage-specific duplication and divergence (Hess and Edwards 2002; Westerdahl 2007).

As the sister group to mammals, reptiles occupy a key phylogenetic position for understanding the evolution of the MHC but have been poorly represented in MHC studies thus far. Nonavian reptiles are represented by four clades: Squamata (lizards and snakes), Rhynchocephalia (tuatara), Crocodylia (crocodilians; birds form a monophyletic group with this clade, Archosauria), and Chelonia (turtles), which together encompass a huge diversity of morphologic, reproductive, developmental, and life history characteristics. These four reptilian clades diverged early in amniote evolution, around 250−280 million years ago (Hugall *et al.* 2007), and thus analysis of MHC structure in nonavian reptiles will fill an important gap in reconstructing the evolutionary history of the amniote MHC. A recent study (Green *et al.* 2014) of MHC organization in the saltwater crocodile (*Crocodylus porosus*) revealed a structure intermediate between eutherian mammals and birds, with larger genes and linkage between class I genes and the framework gene *TRIM39* as in mammals but also linkage between class I and TAP genes as in birds (Jaratlerdsiri *et al.* 2014a). Although additional reptile genome projects are now complete or underway (Alfoldi *et al.* 2011; Castoe *et al.* 2013; Shaffer *et al.* 2013; Wang *et al.* 2013; N. Gemmell, personal communication), the organization of the MHC of nonavian reptiles at genomic level is still poorly known.

Tuatara are the sole extant representatives of Rynchocephalia (also known as Sphenodontia), which diverged from other reptiles around 270 million years ago (Hugall *et al.* 2007). The tuatara genome is unusually large (∼5 Gb) compared with other reptile genomes (Janes *et al.* 2008), and a BAC library (Wang *et al.* 2006) has revealed high repeat content and diversity (Shedlock 2006) and high GC content (Wang *et al.* 2006). A karyotype (Norris *et al.* 2004) and a low-density cytogenetic map of tuatara was facilitated by the BAC library and cDNA clones (O’Meally *et al.* 2009). Previous studies on the tuatara MHC, which included isolation of expressed class I and II MHC sequences from a peripheral blood mononuclear cell library (Miller *et al.* 2005, 2006), analysis of inheritance of class I alleles (Miller *et al.* 2007), and surveys of MHC diversity (Miller *et al.* 2010), suggested that tuatara have at least three class I MHC genes and at least four class II genes. One of the class I genes appears to be either nonclassical or a pseudogene, because it exhibits low nucleotide diversity and is not expressed in peripheral blood mononuclear cells (Miller *et al.* 2007). Two families of class II B genes have been isolated from tuatara cDNA: the SppuDAB family contains at least three genes, and SppuDBB is represented by a single sequence and may be a nonclassical class II gene (Miller *et al.* 2005).

In this study, we aimed to investigate the organization and cytogenetic position of MHC genes in the tuatara genome by identifying, sequencing, and mapping class I and II MHC genes from the tuatara BAC library. Aside from Wang *et al.* (2006), this study therefore represents the first targeted interrogation of a multigene family in the tuatara and provides key insights into the evolution of the MHC in nonavian reptiles.

## Materials and Methods

### Probes and BAC library screening

Probes were constructed by polymerase chain reaction (PCR) amplification of exons 1−5 of class I and II cDNA sequences previously isolated from a tuatara peripheral blood mononuclear cell cDNA library (Miller *et al.* 2005; Miller *et al.* 2006). A class I probe was produced from the Sppu-U*01 cDNA clone (Genbank accession no. DQ145788), and two different class II probes were produced from the Sppu-DAB*01 (DQ124231) and Sppu-DBB (DQ124233) cDNA clones. Primer sequences and PCR conditions for construction of probes is given the Supporting Information, File S1.

High-density filters from the tuatara BAC library VMRC12 (Wang *et al.* 2006), with 6.3× coverage of the tuatara genome, were screened with the class I, class II DAB, and class II DBB probes. Positive clones identified by colony hybridization were further screened by Southern blotting to confirm the presence of class I and/or class II genes. To determine the gene content of clones that showed positive hybridization to either class I or class II probes, PCR amplifications with primers designed to exon 2 of class I genes and exons 2 and 3 of class II genes were performed from purified BAC DNA (Table S1). PCR products were sequenced on an ABI3730 Genetic Analyzer, then sequences were edited using Sequencher 4.2 (GeneCodes Corporation) and aligned with tuatara sequences in the Genbank database using ClustalW implemented in Geneious version 4.6 (Kearse *et al.* 2012, http://www.geneious.com). Further details of hybridization and PCR conditions are given in the File S1.

### BAC fingerprinting

To identify overlapping BAC clones, high-resolution agarose gel fingerprinting of DNA digested with *Eco*RI/*Eco*RV (Marra *et al.* 1997) was performed by the Genome Sciences Center, British Columbia Cancer Agency, Canada. Contigs were assembled with FPC with a tolerance of 7 and cutoff of 1e^-12^ and visualized using Internet Contig Explorer v3.5 (Fjell *et al.* 2003).

### Chromosome mapping

Blood samples were collected from captive animals, which originated from Stephens Island but were held at Taranga Zoo, Sydney (RFID implant numbers 6306A5 and F75DAE). Chromosomes were prepared from short-term culture of peripheral blood leukocytes as described in O’Meally *et al.* (2009). To prepare probes, BAC DNA was purified and labeled by nick translation incorporating either Orange or Green-dUTP (Abbott Molecular). Fluorescence *in situ* hybridization, including the addition of boiled gDNA to suppress repetitive sequences, and visualization of chromosomes was performed as described in O’Meally *et al.* (2009).

### BAC sequencing

BAC clones were sequenced by Amplicon Express using an Illumina HiSeq 2000 and TruSeq SBS v3-HS and TruSeq PE Cluster v3-cBot-HS chemistry. Multiple sizes of paired end and mate pair libraries were sequenced to produce paired 100-bp reads with insert sizes ranging from 200 bp to 7 kb. These data were assembled using Amplicon Express’ proprietary in-house assembly pipeline.

### Gene prediction

Genes were predicted on the assembled BAC contigs >2000 bp using Genscan (Burge and Karlin 1997) and Maker (http://www.yandell-lab.org/software/mwas.html). Repeatmasker (Smit *et al.* 2011) was used to identify and mask repetitive elements prior to running Genscan. Genscan was run via the Pasteur Institute web portal (http://mobyle.pasteur.fr/cgi-bin/portal.py?#forms::genscan), and the output file was converted to GFF format using a custom perl script. MAKER was run via the Web Annotation Service (http://www.yandell-lab.org/software/mwas.html) with the tuatara transcriptome dataset from Miller *et al.* (2012) and an *Anolis carolinensis* protein dataset from Ensembl (AnoCar2.0.72) as expressed sequence tag and protein evidence, respectively. The software Augustus (Stanke *et al.* 2008) was used for *ab initio* gene prediction.

Genscan and Maker outputs in GFF format were imported into Geneious R7 (Kearse *et al.* 2012, http://www.geneious.com) and loaded onto the assembled contigs. All predicted exon domains were then BLASTed against the Genbank database using tblastx with an E-value cut-off of 1 × 10^−3^. Additional searches for MHC genes were performed by using the “Annotate from Database” function in Geneious, which used a BLAST-like algorithm to match known tuatara MHC sequences from cDNA (Miller *et al.* 2005, 2006) to the BAC clone sequence. Where an MHC gene was predicted, the exon boundaries were confirmed by extracting the predicted gene and aligning it to tuatara MHC cDNA sequences SppuU*01 for class I (Miller *et al.* 2006) or SppuDAB*01 for class II (Miller *et al.* 2005), then manually editing the exon boundaries as required. Genes were named according to established MHC nomenclature, with the first two letters of the genus and species name (Sppu), followed by letter/number combinations denoting the locus. Class I sequences were divided into UA, UB, and UC groups based on how they clustered on a phylogenetic tree. For class II sequences, all sequences that clustered with existing SppuDAB cDNA sequences were named SppuDAB, and then given a number (*e.g.*, 01, 02). The suffix “bac” is added to these sequences to distinguish them from previously isolated cDNA sequences. Because the cDNA library and BAC libraries were created from different animals and each may be heterozygous, it is not possible to determine how these sequences are related and no further attempt has been made to classify them into orthologous loci. More divergent class II sequences were named SppuDBB, DCB, and DDB.

Gene density for the tuatara MHC was approximated by the use of scaffolds greater than 10,000 bp long from clones that map to chromosome 13. The total number of genes, excluding partial genes, ribosomal genes, retrotransposons, and repetitive elements, was divided by the total number of base pairs in the scaffolds. The clone mapping to chromosome 4 was not included in this analysis as this is likely to be out of the core MHC region.

### Phylogenetic analysis and adaptive evolution of tuatara MHC genes

Putative coding regions for MHC genes were extracted and aligned using MUSCLE, as implemented in Geneious R7. For class I genes, exons 3 and 4 were used for phylogenetic analysis, and for class II genes, exons 2 and 3 were used. Sequences for comparison were downloaded from Genbank and are shown according to the first two letters of the genus and species name, plus the gene name (See Table S5 and Table S6 for accession numbers). Sequences previously isolated from tuatara cDNA also were included. Maximum likelihood trees were constructed in PHYML (Guindon and Gascuel 2003) with the GTR+G+I model with 500 bootstrap replicates, and 50% majority rule trees were then built using the Geneious consensus tree builder. For class II genes we first analyzed all available reptile class II sequences for which exons 2 and 3 were available, including the nonclassical *DM* genes and genes from reptile genome builds available at the National Center for Biotechnology Information. *DM* genes and some class II sequences from *Chrysemus picta* and *Chelonia mydas* genomes were highly divergent from all other reptile class II genes and could not be reliably aligned; therefore, they were removed before construction of the final tree.

We measured adaptive evolution of tuatara class I and II genes in PAML version 4.4 (Yang 2007) by using the sites models in the codeml software and Bayes Empirical Bayes criterion to identify putative adaptively evolving sites (Yang *et al.* 2005). For both classes, we compared the likelihood of model 7 (no adaptive evolution) to model 8 (including putative adaptive sites) by using a likelihood ratio test, with rate variation among sites and pairwise removal of sequence gaps. This analysis was performed on all BAC-derived and previously identified cDNA sequences from tuatara, with the exception of those containing out of frame insertions (see Figure S2 and Figure S3). Sequences removed were SppuDAB-06bac for class II, and SppuUCB and SppuUD for class I. However, other putative pseudogenes were included, as pseudogenes may still display evidence of past selection if pseudogenization has been recent (Miyata and Yasunaga 1981, Zhang 2014) or may even exhibit signatures of reactivation, as has been found in human MHC genes (Doxiadis *et al.* 2006).

## Results

### Isolation of BAC clones

Probes for class I, class II DAB, and class II DBB loci were used to screen the tuatara BAC library, resulting in the isolation of 84 clones. Of these, 71 were confirmed by Southern blotting to hybridize to either class I or class II probes, or both (Table S2). Forty-seven of these clones assembled into 11 fingerprint contigs, each containing between 2 and 9 clones (Figure S1). Twenty-four clones did not assemble into contigs.

PCR amplification using primers designed from MHC class I and II cDNA sequences was used to confirm overlaps within contigs and to look for the presence of classical class I and II genes. Class I sequences matching the previously isolated class I alleles U*11 (2 clones) and U*19 (4 clones) (Miller *et al.* 2007) were amplified from fingerprint contig 4 and 3 single clones. However, no class I sequences could be amplified from the remaining 31 clones that hybridized with the class I probe, suggesting that these clones contain more distantly related class I−like genes. Multiple different class II DAB sequences were amplified from fingerprint contigs 4, 6, 7, and 8, plus eight single clones, and a single DBB sequence was amplified from the six clones comprising fingerprint contig 1. Fingerprint contig 4 and the single clone 531J19 contained both SppuDAB and classical class I sequences. Twelve clones hybridized with class II probes but did not contain Sppu-DAB or DBB sequences. For full details of clones and PCR markers contained in each fingerprint contig, see Table S2.

### Cytogenetic mapping

**Figure 1:**
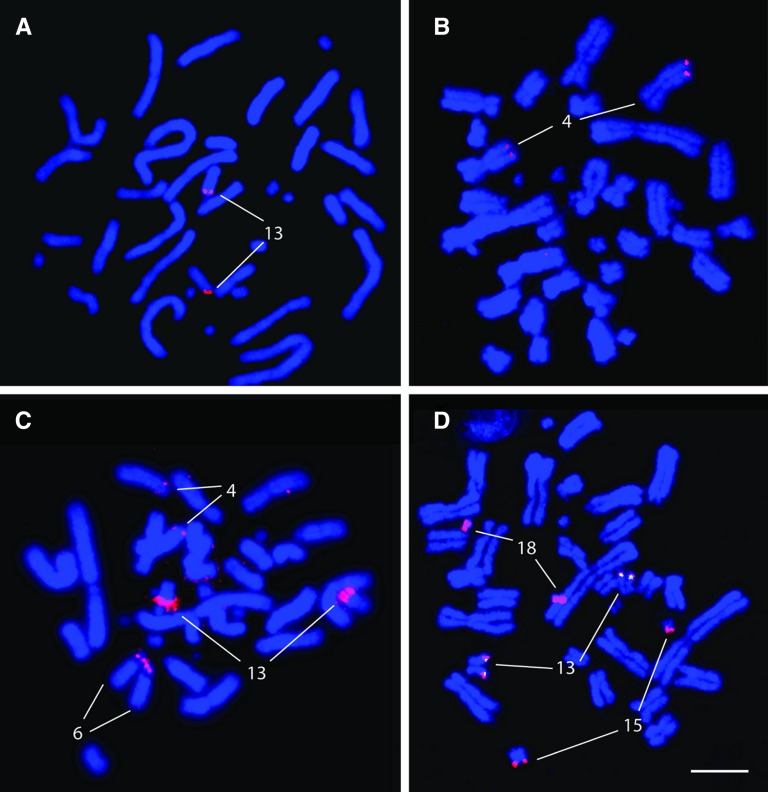
Fluorescence *in situ* hybridization of major histocompatibility complex−containing bacterial artificial chromosome clones on tuatara metaphase chromosomes. (A) Clone 534N11 hybridizing to chromosome 13q; (B) 44B3 hybridizing to chromosome 4q; (C) 448I11 hybridizing to chromosomes 4q, 6p, and 13q; (D) colocalization of 500B16 (red) and 346M2 (green) on chromosome 13q. 500B16 also hybridizes to 2 microchromosomes (15 and 18).

One BAC clone each from fingerprint contigs 1, 2, 4, 6, 7, and 8 plus individual clones 531J19, 534N11, 342M2, 437A11, and 500B16 were mapped to tuatara metaphase chromosomes using fluorescence *in situ* hybridization (Figure 1). These clones were chosen because they contained SppuDAB, SppuDBB, and/or class I sequences, or displayed strong hybridization with the class I probe. Clones from contigs 3, 5, 9, 10, and 11 were not mapped because they only hybridized weakly to class II or class I probes in Southern blotting and no MHC genes could be isolated from them using PCR.

**Figure 2:**
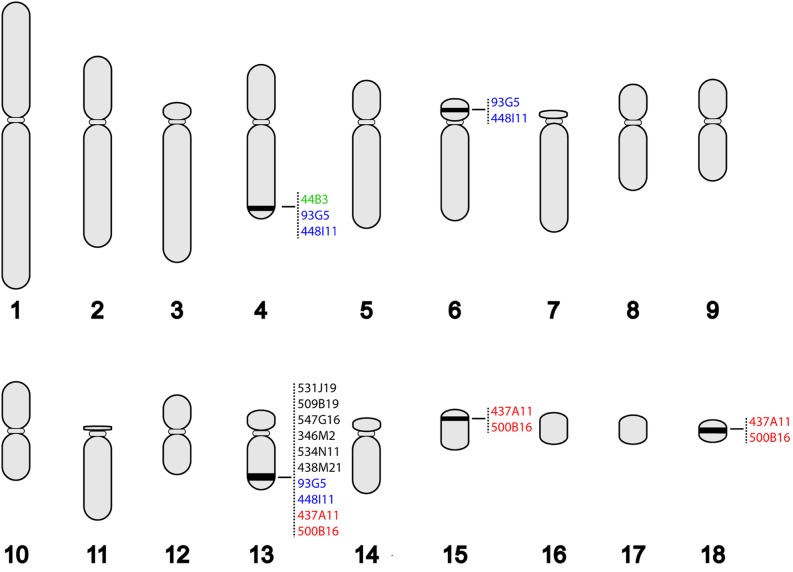
Karyogram of tuatara showing the location of bacterial artificial chromosome (BAC) clones mapped in this study. Colors indicate the hybridization pattern of BAC clones that mapped to more than one chromosome.

All clones except 44B3 were assigned to chromosome 13q, but four of these clones mapped to other chromosomes as well (Figure 2). The clones from contigs 7 and 8, 93G5 and 448I11 respectively, were assigned to chromosomes 4q and 6p as well as chromosome 13q, and clones 437A11 and 500B16 were assigned to two microchromosomes in addition to chromosome 13q. Clone 44B3, which represents fingerprint contig 2 and contains class I genes, hybridized only to chromosome 4q. These results suggest a core MHC region is contained on chromosome 13q, but there may also be duplicated regions on chromosomes 4 and 6 as well as on 2 microchromosomes.

### Characterization of tuatara BAC clones

The same BAC clones that were mapped to chromosomes were sequenced using paired-end Illumina sequencing. Because multiple class I and II genes were identified by PCR on fingerprint contig 4, an additional clone from this contig, 553D12, was sequenced to provide complete coverage of the contig. BAC clone assemblies were somewhat fragmented, with 1−13 scaffolds assembled per clone. The percentage of missing data (Ns) in each clone ranged from 0.7 to 19.3% (see Table S3 for assembly statistics). Scaffolds larger than 2000 bp were annotated using Genscan, MAKER, and blastx, and with the exception of clone 346M2, the MHC genes expected from probe hybridization and/or PCR screening were found. Clone 346M2 hybridized to the MHC class II probe and a PCR product was obtained with class II exon 3 primers, but no MHC genes or MHC-associated genes could be identified on this clone, suggesting that this was a false-positive result or that the sequencing was insufficient. This clone appears to contain only zinc-finger BED domain-containing sequences and transposable elements such as CR1-3 homologs, which frequently are found near MHC genes in birds and other vertebrates (Gasper *et al.* 2001; Shedlock 2006).

Each BAC clone sequenced contained between one and five genes or partial genes, fewer than are located on MHC-associated BAC clones sequenced in other species (Table 1). Tuatara BAC clones that map to the likely core MHC region on chromosome 13 average one gene per 66.7 kb (0.015 genes per kb), which is lower than that observed in zebra finch (0.047 genes/kb), chicken (0.111 genes/kb), and human (∼0.021 genes/kb) (Balakrishnan *et al.* 2010). All clones contained numerous reverse-transcriptase-like elements and repetitive elements (see Table S4), and many also contain zinc-finger domains. The frequency of long interspersed nuclear elements (LINEs), mainly CR1 and L2 elements, is exceptionally high, at 0.39 per kb, as compared with 0.07 per kb in zebrafinch and 0.02 per kb in chicken. The frequency of long terminal repeats is 0.07 per kb, compared with 0.14 per kb in zebrafinch and 0.01 per kb in chicken.

**Table 1:** Chromosomal locations and gene content of BAC clones mapped and sequenced in this study

Clone (Contig)	Chromosome	MHC Genes
438M21 (1)	13	Class II beta SppuDBB (full length)
509B19 (4)	13	Class I SppuUBA (U*19, full length)
		Class I SppuUBB (partial, exons 3-6)
		Class II beta SppuDAB-01bac (missing part exon 1*)
		Class II beta SppuDAB-03bac (full length)
		Class II beta SppuDAB-04bac (exons 1, 3, and 6 only, pseudogene?)
		Class II alpha SppuDAA-1 (partial, exons 2-4)
553D12 (4)	13	Class I SppuUBA (U*19, full length)
		Class II beta SppuDAB-01bac (missing part exon 1*)
		Class II beta SppuDAB-02bac (69 bp deletion in exon 2)
		VWA5A (partial)
531J19	13	Class I SppuUAA (U*11, full length)
		Class II beta SppuDAB-08bac (2 fragments)
547G16 (6)	13	Class II beta SppuDCB (full length)
		SPTLC1 (processed pseudogene)
		3 zinc-finger related loci
534N11	13	Class II beta SppuDAB-06bac (partial, exons 1-5, 11 bp deletion in exon 2)
346M2	13	None
93G5 (7)	4+6+13	Class I SppuUDA (partial, exons 2-4)
		Class II beta SppuDAB-05bac (partial, missing exon 2*)
		2 zinc-finger related loci
448I11 (8)	4+6+13	Class II beta SppuDDB (partial, exons 3 and 4)
		SCARA5 (partial)
437A11	13+µ	Class II alpha SppuDAA-2 (partial, exons 1, 3 and 4)
500B16	13+µ	Class II SppuDAB-07bac (partial, missing exon 2)
		Class II alpha chain (partial, exon 4 only)
44B3 (2)	4	Class I SppuUCA (full length, no stop codon?)
		Class I SppuUCB (full length, pseudogene)
		Class I SppuUCC (partial, exons 3-6)

“Contig” refers to fingerprint contigs (see Figure S1). Class I sequence variants are named according to Miller *et al.* (2006). Genes marked with an asterisk (*) are those where missing exons correspond to missing sequence in the assembly. BAC, bacterial artificial chromosome; MHC, major histocompatibility complex.

**Figure 3:**
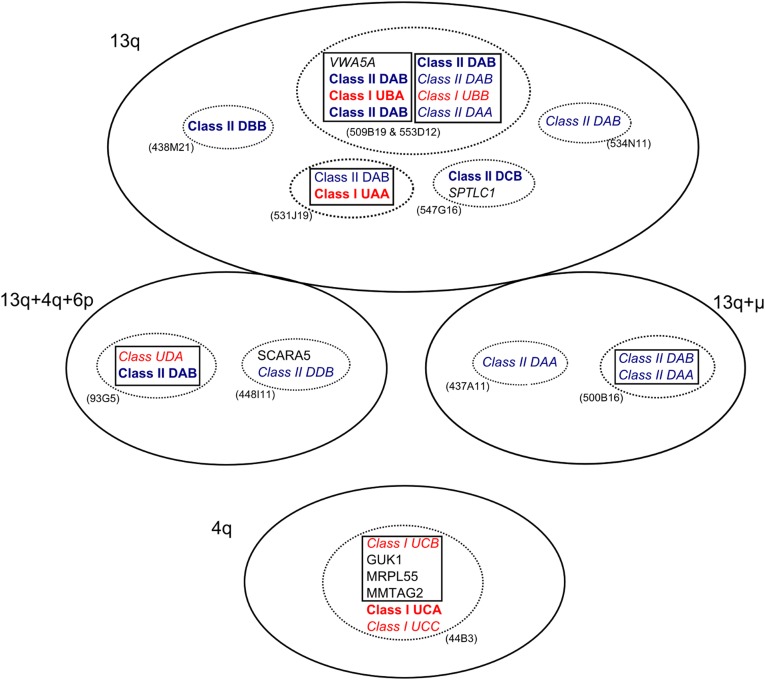
Organization of genes on bacterial artificial chromosome (BAC) clones mapped in this study. Genes in boxes are on the same BAC scaffold, and genes in dotted ovals are on the same BAC clone or fingerprint contig but may be on different scaffolds. BAC clone IDs are given in parentheses next to each oval. BAC clones that map to the same chromosome are shown in the same solid oval with the chromosomal location given. Class I genes are in red, and class II genes in blue. A gene name in bold indicates a full-length gene (or one where a missing segment is probably due to sequencing artifact), and genes in italics are putative pseudogenes, or partial genes. Predicted zinc-finger domains are not shown. For further details of genes in each BAC clone, refer to Table 1.

Clones mapping solely to chromosome 13 contained classical class I as well as class II α and β genes, further suggesting that the core MHC region is found here (see Figure 3 and Table 1). Individual scaffolds from the two clones which span fingerprint contig 4 (509B19 and 553D12) could be merged into two scaffolds spanning the entire contig. The first scaffold is 163,900 bp and contains a full-length class I gene identical to allele U*19 in exon 2 (Miller *et al.* 2007) and two class II β genes. The class I gene lies between the two class II β genes, suggesting these genes are intermingled rather than in discrete regions. It is not clear whether the class II β genes (named SppuDAB-01bac and SppuDAB-02bac) represent functional genes. SppuDAB-01bac is missing part of exon 1 but otherwise appears functional. Both 553D12 and 509B19 have a stretch of missing data in the region that should contain the missing part of exon 1, suggesting that the missing sequence is an assembly artifact. SppuDAB-02bac has a deletion of 69 bp in exon 2. The 5´ end of this scaffold also contains the last 3 exons of a von Willebrand factor A domain-containing protein (*VWA5A*). This gene maps to chromosome 11 in humans, outside of the human MHC.

The second scaffold from fingerprint contig 4 contains a full-length class II β gene named SppuDAB-03bac, and a second partial class II β gene (SppuDAB-04bac). SppuDAB-04bac appears to be missing exons 2, 4, and 5 but has high similarity with the SppuDAB*01 cDNA in the remaining exons. A partial exon 2 sequence is located 1050 bp upstream of exon 1. Thus, either this gene is pseudogene or there has been a misassembly of the scaffold in this region. This scaffold also contains a partial class II α chain gene, and a class I gene fragment, which comprises only exons 3-6, contains a frameshift deletion in exon 4 and a stop codon in exon 5. The class II α gene is located at the extreme 3´ end of the scaffold and contains exons 2−4, suggesting that exon 1 of this gene is located outside the scaffold. Additional partial class II α chain genes were found on clones 437A11 and 500B16, which map to chromosome 13 and two microchromosomes.

Clone 531J19, which is not part of a fingerprint contig but maps to chromosome 13, also contains both class I and class II genes. The class I gene on this clone is full length and identical to the U*11 allele in exon 2. Two fragments of a class II DAB-like sequence were also present on scaffold 1 of this clone. The first fragment contains exons 3-6 plus the 3′UTR, and the second contains exons 1−4. These fragments are identical in their region of overlap, exons 3 and 4, and thus may in fact be two parts of the same gene, suggesting that the scaffold is misassembled. A stretch of missing data (>5000 Ns) between exons 2 and 3 in the second gene fragment lends weight to this suggestion. For the purposes of further analysis, the two fragments were merged into a single gene, named SppuDAB-08bac.

Additional class II β sequences were found on clones 438M21, 547G11, and 534N11, which map solely to chromosome 13, and on 93G5, 448I11, and 500B16, which map to multiple chromosomes including chromosome 13 (Figure 3). The class II β sequences on 93G5 and 500B16 are DAB-like, but are missing exon 2. The sequence on 93G5 (SppuDAB-05bac) has missing sequence in the region where exon 2 would be located, suggesting the missing exon is the result of an assembly artifact. The 93G5 clone also contains a class I gene (SppuUD) which is highly divergent from previously isolated classical class I sequences; only exons 2−4 appear class I-like and exons 1, 5, and 6 (identified by Genscan) bear little resemblance to known class I genes. The class II β gene on clone 547G11 is full length, but divergent from both DAB and DBB genes and has been named SppuDCB. The class II β sequences found on 534N11 and 448I11 are partial genes. Clone 438M21 contains a single, full length MHC class II gene matching the SppuDBB cDNA sequence (Miller *et al.* 2005). In total, 11 different class II β sequences were isolated across nine BACs (Table 1), but at least five are gene fragments or contain indels or premature stop codons suggesting they are nonfunctional.

Additional class I genes were found on the clone mapping to chromosome 4, 44B3 (fingerprint contig 2). This clone contains two full-length class I genes, at least one of which may be a pseudogene due the presence of a stop codon in exon 2 and a single base pair deletion in exon 3. Additionally, the clone contains exons 3−5 of a third class I MHC gene. These sequences were divergent from the class I sequences mapping to chromosome 13 and were named SppuUCA, UCB, and UCC. Scaffold 1 of this clone also contains 3 non−MHC-associated genes: *GUK1*, *MRPL55*, and *MMTAG2* (C1orf35). The region containing these genes lies on chromosome 1 in humans (1:228,100,726–228,148,984), chromosome 2 in chicken (2:2,334,607−2,358,790) and chromosome 6 in the *Anolis* lizard (6:3,238,928−3,275,679).

### Comparative analysis of tuatara MHC genes

**Figure 4:**
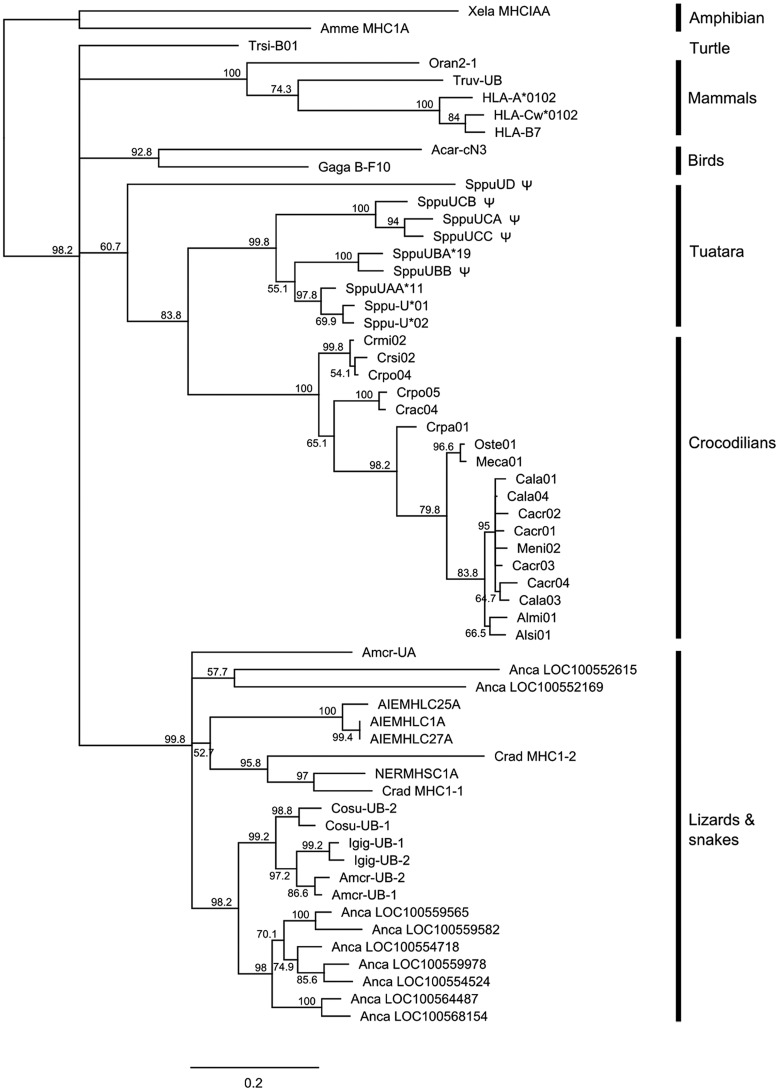
Maximum likelihood tree of class I exon 3 and 4 sequences, with 500 bootstrap replicates. The tree is drawn as a 50% majority rule consensus tree. Putative pseudogenes are marked with ψ.

A phylogeny of class I and II sequences isolated from tuatara BAC clones in relation to other reptiles was generated with the use of maximum likelihood. The tuatara class I sequences (Figure 4) form a single clade, with the exception of the SppuUD sequence isolated from clone 93G5. Within the main tuatara clade are three subclades: one containing the sequences isolated from cDNA, plus the SppuUAA sequence from 531J19; a second containing the UB sequences from BAC contig 4, and a third containing the SppuUC sequences from 44B3 that map outside the putative core MHC region. MHC sequences within each reptilian order cluster together with strong bootstrap support, but relationships among orders are not resolved in a 50% majority rule consensus tree. The tuatara sequences appear most closely related to crocodilian sequences, but the branches separating the two clades are long compared to the branch lengths between sequences within each clade and in the absence of additional taxon sampling, long branch attraction cannot be ruled out (Felsenstein 1978).

**Figure 5:**
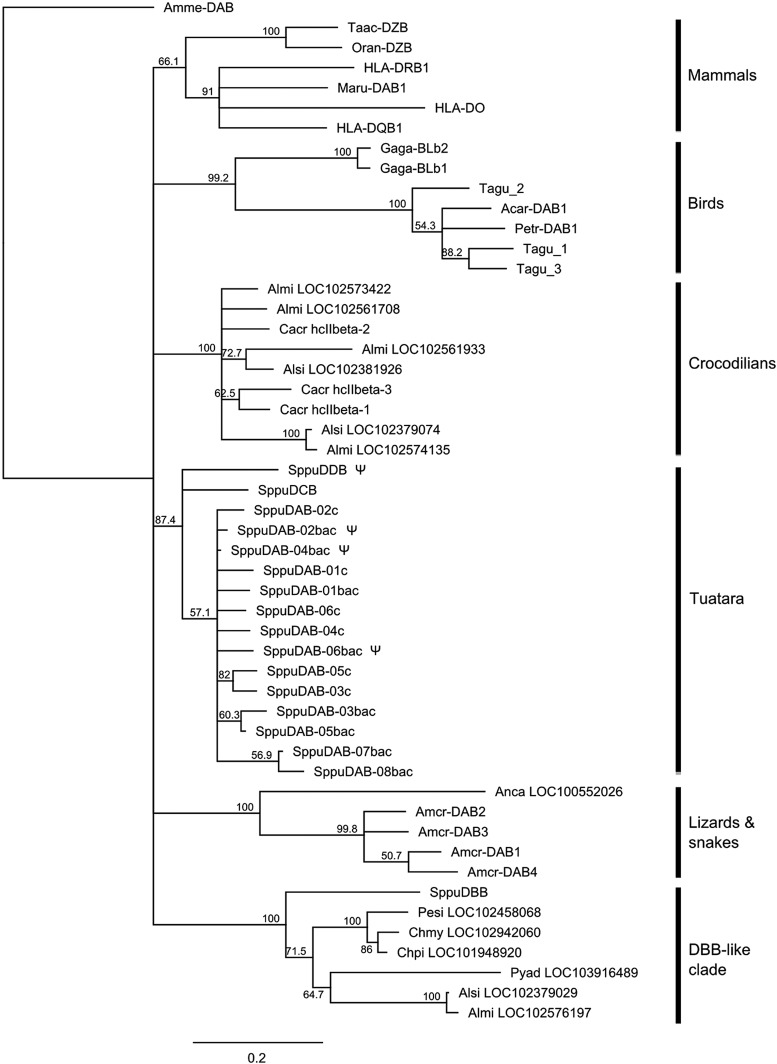
Maximum likelihood tree of class II exon 2 and 3 sequences, with 500 bootstrap replicates. The tree is drawn as a 50% majority rule consensus tree. Tuatara DAB sequences isolated from cDNA are denoted by the suffix “c” and those isolated in this study with the suffix “bac.” Putative pseudogenes are marked with ψ.

The tuatara class II sequences also form a single clade, with the exception of the SppuDBB sequence from clone 438M21 (Figure 5). The putative SppuDAB sequences form one clade that includes the DAB sequences previously isolated from cDNA (Miller *et al.* 2005), but relationships within the clade are not well-resolved, and it is not possible to determine which sequences represent orthologous loci. The SppuDCB and DDB sequences fall outside the DAB clade but are still more similar to these tuatara sequences than to sequences from other reptiles. The SppuDBB sequence clusters with 100% bootstrap support in a clade containing sequences from the turtles *Pelodiscus sinensis*, *Chelonia mydas*, and *Chrysemus picta*; the crocodilians *Alligator mississippiensis* and *Alligator sinensis*; and Adelie penguin *Pygoscelis adeliae*. Only a single sequence from each of these species fell into this clade: a separate clade of crocodilian sequences, sister group to the avian class II sequences was also present; other predicted class II-like sequences from the genome builds of the turtles *Chelonia mydas* (Wang *et al.* 2013) and *Chrysemus picta* (Shaffer *et al.* 2013) were so highly divergent from other reptile class II sequences that they were omitted from the tree. Aside from the SppuDBB clade, class II sequences from reptiles cluster by order, but as with the class I sequences, the relationship among orders is not strongly supported.

We searched for evidence of adaptive evolution in tuatara MHC genes using PAML. A total of six sites in the class I alignment and 12 sites in the class II gene alignment were deemed adaptively evolving by the Bayes Emprical Bayes criterion in PAML (Table S7, Figure S2, and Figure S3). Five of the class I sites and 11 of the class II sites are putative peptide binding sites, based on the structures of the human leukocyte antigen HLA-A and DRB1 molecules, respectively (Bjorkman *et al.* 1987, Brown *et al.* 1993). These sites have been identified as adaptively evolving in other studies (*e.g.*, Burri *et al.* 2008; Glaberman *et al.* 2009; Balakrishnan *et al.* 2010). Overall, for both class I and II, there was significant evidence for a model including adaptively evolving sites (−lnL = −2945.5 and−3060.6, respectively) compared with a model without adaptive evolution (lnL = −2969.5 and −3109.6, respectively; *P* < 0.001, df = 2).

## Discussion

The results presented here add a critical lineage to the emerging picture of MHC evolution in amniotes, with a genome-level characterization of MHC organization in an evolutionarily divergent reptile, the tuatara. The tuatara MHC region appears to be large with a high repeat content. We found a total of seven class I sequences and 11 class II β sequences, but some appeared to represent pseudogenes. Chromosome 13q appears to contain the core MHC, as clones containing classical class I, class II beta, and class II alpha chain genes map to here, but additional class I genes were located chromosome 4p.

The MHC in tuatara has low gene density compared with other species. We found at most five genes on individual BAC clones, and many clones contained only one or two genes and a high number of repetitive elements. The low density of tuatara MHC genes is a likely reason for challenges in identifying other MHC-associated genes like *TAP1*, *TAP2*, *TAPBP*, or *C4* or framework genes like *DAXX*, *BRD2*, or *TNXB* on these BACs. The non-MHC genes we identified that map to chromosome 13—*VWA5A*, *SCARA5*, and *SPTLC1*—are not found in the MHC in other species. However, we could not confirm whether the copies isolated here are functional, because we did not find the complete coding sequences for any of these genes. The low gene density appears to be a feature of the tuatara genome in general, as the region from *GUK1* to *C1orf35* on clone 44B3 that maps to chromosome 4 spans 82,100 bp in tuatara, whereas the orthologous regions in human is 48,258 bp, in *Anolis* is 36,750 bp, and in chicken spans only 24,183 bp. Other low-density regions of the tuatara genome involving the *DMRT1* gene have also been identified (Wang *et al.* 2006).

The accumulation of repetitive elements is likely to be responsible for the increased intergenic distances and long introns observed in tuatara and in reptiles generally, and underlie its large genome size of approximately 5 Gbp (Shedlock 2006; Organ *et al.* 2007; Janes *et al.* 2010). We found a high diversity of repeats in the tuatara BAC clones sequenced here (Table S4), including a particularly high number of LINEs, mainly CR1 and L2 retroelements. This pattern also was observed by Wang *et al.* (2006) and Shedlock (2006), who found that tuatara had more than twice the number of repeat types per megabase than *Anolis* and the greatest number of LINE retroelements of any of the reptiles in their study. A number of clones also contained zinc-finger protein domains, similar to that observed in the passerine MHC (Edwards *et al.* 2000; Gasper *et al.* 2001; Balakrishnan *et al.* 2010). This high repeat content also may have contributed to assembly problems in some clones, as almost all contained missing sequence and multiple scaffolds. Clone 531J19 in particular may have been misassembled, as the class II gene on this clone was in two fragments, with a duplicate exon 3 and 4. Future genome assembly projects for tuatara will require strategies for overcoming the highly repetitive nature of the genome, such as the use of long read sequencing technology (*e.g.*, Eid *et al.* 2009).

Retroelements also may have been responsible for the hybridization of some MHC-containing BAC clones to multiple chromosomes. All of the class II sequences map to chromosome 13q, but some of the clones containing these loci cohybridize with two microchromosomes or chromosomes 4q and 6p as well as chromosome 13. Despite the use of suppressive DNA in the probes, it is possible that repetitive elements in these clones are responsible for their cohybridization to multiple chromosomes, rather than a duplication of the MHC region itself. A similar result was seen in zebra finch, where class II BACs hybridized to several pairs of microchromosomes each, probably because of shared repeat content of the clones (Balakrishnan *et al.* 2010).

The class I genes identified in our study fall into four clusters in phylogenetic analysis and map to two distinct locations. The UA and UB sequences map solely to chromosome 13q, while UC sequences map to chromosome 4q. Because these sequences are located on separate BAC clones that map to single chromosomes, they are likely to represent a real duplication and translocation event rather than an artifact caused by repetitive elements. A single UD sequence, which may represent a nonfunctional fragment, is on a clone mapping to chromosome 13q and microchromosomes. The finding of MHC genes on more than one chromosome has only been observed in a handful of other species. In the tammar wallaby, classical class I genes appear to be spread across multiple chromosomes, away from a core MHC region containing the class II, class III, antigen-processing, and MHC framework genes (Deakin *et al.* 2007; Siddle *et al.* 2011). In teleost fish, class I and class II genes are found on different chromosomes (Sato *et al.* 2000; Kuroda *et al.* 2002) and in zebra finch, MHC-containing BAC clones mapped to two different chromosomes (Balakrishnan *et al.* 2010), although the classical class I and class II genes mapped to a single chromosome (Ekblom *et al.* 2011). These findings have refuted the hypothesis, put forward by earlier studies of MHC comparative genomics (*e.g.*, Kelley *et al.* 2005), that colocalization of MHC genes in one region is necessary for function. However, the tuatara class I genes that map to chromosome 4 are unlikely to be classical class I genes. They are divergent from class I sequences isolated from cDNA and do not contain all of the conserved residues expected in classical class I genes (Kaufman *et al.* 1994). The UCA and UCB genes are full length, but the UCA gene may be missing a stop codon and UCB has a stop codon in exon 2 and a single base-pair deletion in exon 3. Only exons 3−6 of UCC were present. Thus, these sequences are likely to be pseudogenes. The UA and UB sequences that map to chromosome 13 are the best candidates for classical class I genes because they have been identified previously as polymorphic from population studies and fall in the same phylogenetic cluster as sequences expressed in peripheral blood mononuclear cells (Miller *et al.* 2006). The other genes that were found on the BAC mapping to chromosome 4 (*GUK1*, *MRPL55*, and *MMTAG2* (*C1orf35*)) are not found in the MHC in human or chicken. Class I genes are not found in the orthologous region in *Anolis*, suggesting that a class I MHC gene was translocated to this region after the split of Rhynchocephalia from other reptiles and then duplicated here. This idea is supported by the fact that UA, UB, and UC sequences fall into a single clade with high bootstrap support on the phylogenetic tree, and the UC sequences form a subclade within this. Neither the UC or UD sequences are homologous to the non-polymorphic and non-expressed “UZ” locus identified in Miller *et al.* (2007).

The presence of multiple class II β sequences in this study is consistent with an earlier study on cDNA in which authors found at least six expressed DAB sequences and one DBB sequence (Miller *et al.* 2005). The cDNA library was constructed from a different individual than was the BAC library, and the DAB sequences from cDNA differ from the sequences isolated in this study but cluster closely in the phylogenetic analysis, with 97.9% mean pairwise sequence divergence in exon 3. The DAB, DCB, and DDB sequences from tuatara form one clade on the phylogenetic tree, with the DAB sequences forming a sub-clade within this. SppuDCB appears to represent a functional class II β chain gene because it does not contain any indels and contains most of the conserved residues expected of classical class II genes (Kaufman *et al.* 1994). In contrast, SppuDDB is likely to be a pseudogene, because only exons 3−5 were present and exon 3 has a frameshift deletion. Some of the DAB sequences also are likely to be pseudogenes: only SppuDAB-03bac has all expected exons with the correct stop codon and no indels. However, missing sequence in three other DAB sequences (DAB-01bac, DAB-05bac, and DAB-09bac) was probably attributable to sequencing or assembly artifacts so these sequences may also represent functional genes. The identification of 12 positively selected sites that correspond with putative peptide binding sites, along with strong support for an adaptive evolution model among the class II sequences, lends weight to the suggestion that many of the class II sequences represent functional, classical loci.

The finding of multiple duplications of class II genes in tuatara contrasts with *Anolis carolinensis*, which appears to only have a single class II beta gene (Alfoldi *et al.* 2011). However, the *Anolis* genome may be unusual: the saltwater crocodile MHC appears to have undergone substantial duplication, with 9 class I and 6 class II genes (Jaratlerdsiri *et al.* 2014a). MHC data for other reptile species is scarce, but in the few species for which class II MHC sequences are available, multiple copies of class II genes appear to be present (*e.g.*, the Galapagos marine iguana, Glaberman *et al.* 2009, and alligators *A. mississippiensis* and *A. sinensis* (St John *et al.* 2012)). Passerine birds are similar to tuatara in the level of duplication and pseudogenization of class II genes (Westerdahl *et al.* 2000, Miller and Lambert 2004, Balakrishnan *et al.* 2010).

The diversity of class I and II MHC genes observed in our study appears to be a classic example of the birth and death model of evolution (Nei *et al.* 1997), in which MHC genes evolve by frequent duplication and pseudogenization. We found evidence for positive selection shaping the diversity of MHC genes in tuatara, but concerted evolution (*e.g.*, Wittzell *et al.* 1999) also may play a role in maintaining clusters of closely related genes (such as the DAB genes in tuatara). Multiple rounds of duplication are evident for both class I and class II genes, with more distantly related sequences (*e.g.*, UA, UB and UC for class I and DAB, DBB, DCB, and DDB for class II) representing older duplication events, and expansions within each group representing more recent events and/or homogenization by concerted evolution. Orthologous lineages are erased over time by concerted evolution, divergence by point mutation, positive selection and gene conversion, and gene loss, and in our analyses both class I and class II genes (with the exception of SppuDBB, see below) clustered strongly within reptilian orders. This finding is unsurprising, as the four reptilian orders have been isolated from one another for 250−300 million years, far longer than the estimated turnover times for MHC genes in other lineages (Takahashi *et al.* 2000; Piontkivska and Nei 2003; Burri *et al.* 2010; Jaratlerdsiri *et al.* 2014b). Gene duplication and loss within each order has led to differences in gene number across reptile species, but in some cases orthologs have been identified between lineages dating back to around 100 MYA (*e.g.*, Burri *et al.* 2010; Jaratlerdsiri *et al.* 2014b). The long time that the major orders of reptiles have been evolving independently and the lack of orthology among MHC genes from different orders makes it difficult to speculate how complex the MHC was in the ancestral amniote, but analyses of MHC organization in other reptile orders will help to identify common structural features.

The clade containing the SppuDBB sequence appears to be the exception to ordinal clustering, because it also includes turtle, crocodile, and bird sequences. Additional class II sequences from these orders fall in separate clades, suggesting two lineages of class II genes are present in reptiles. As discussed by Miller *et al.* (2005), SppuDBB contains a number of substitutions in the peptide binding and CD4 binding regions and may be a nonclassical class II gene, but it bears little similarity to known nonclassical class II genes such as *DM* and does not cluster with *DM* genes on a phylogenetic tree (data not shown). The SppuDBB sequence from the BAC clone is almost identical to the DBB cDNA sequence, differing only at 3 base positions, yet comes from a different individual. This finding suggests that this locus exhibits only low levels of polymorphism, but sequencing of additional individuals will be required to confirm this. This sequence may represent an ancient lineage of non-classical class II genes in reptiles. Although an ortholog has not yet been found in squamates, which are thought to be the sister group of tuatara, its presence in the more distantly related crocodilian and turtle lineages suggests that it may represent the ancestral reptilian condition. Further genome sequencing will help to confirm whether this is the case.

The lack of MHC framework and antigen processing genes in the BAC clones we isolated makes it difficult to draw any definite conclusions about large-scale MHC structure in tuatara. We would expect the core structure to be similar to that observed in birds, with adjacent class I and class II regions and TAP genes within the class I region. We did observe evidence that some class I and II genes are intermingled, rather than lying in discrete regions. For example, the putative classical class I genes in tuatara are found in close proximity to class II genes, with SppuUAA being adjacent to SppuDAB08 on 531J19 scaffold 1 and SppuUBA found in between SppuDAB01 and SppuDAB02 on contig 4 scaffold 1. It is unusual to find class I and class II genes so close together without any intervening genes, and this could be the result of low levels of interlocus gene conversion in the tuatara MHC. Of the MHC genomic structures so far determined, only the opossum MHC shows some intermingling of class I and class II genes (Belov *et al.* 2006). In galliform birds (the only avian lineage in which MHC organization has been fully characterized), the class I and class II regions are adjacent but separated by *DM* and *BRD2*/*RING3* genes (Kaufman *et al.* 1999, Shiina *et al.* 2004, Wang *et al.* 2012), neither of which were found in the tuatara BACs we sequenced. In the saltwater crocodile, MHC class I and II genes occur on separate contigs (Jaratlerdsiri *et al.* 2014a).

Another difference in MHC organization between birds and mammals is in the placement of class II α chain genes. In mammals, α and β chain genes are found in pairs (although some expansion of β chain genes within gene families has occurred), whereas in chicken a single α chain gene is located away from the core MHC region (Kaufman *et al.* 1999). In tuatara, partial class II α chain genes were found on the same BAC scaffold as class II β genes, indicating that they are within the core MHC as in mammals, although there was no obvious pairing of α and β chain genes. At least two of the three class II α genes may be pseudogenes as they were missing exons. The SppuDAA-1 locus on contig 4 is the best candidate for a functional α chain gene as it contains complete exons 2−4, and the missing exon 1 is likely to be located off the end of the contig.

This study represents a first step in understanding the structure of the MHC in tuatara, the sole representative of an early-diverging order of reptiles. We found some key features that separate the tuatara MHC from that of birds, the only group falling within the Sauropsida for which the MHC genomic structure has been characterized in detail. Like the tuatara genome itself, the MHC region is large and characterized by a high repeat content. Multiple gene duplications, pseudogenization, intermingling of class I and class II genes, and translocation of some class I genes away from the core MHC region, point to a highly dynamic MHC that probably bears little resemblance to the ancestral reptilian MHC. These findings, compared with mammalian and bird MHCs, show that the MHC has undergone substantial change across the major amniote lineages since they began to split approximately 310 million years ago. The organization of the tuatara MHC appears to differ from the few non-avian reptiles examined, but whether its organization is typical of other extant reptiles is unknown. Our results highlight the need for high-quality annotation of MHC regions of newly-sequenced reptile genomes.
